# An examination of exposure and avoidance behavior related to second-hand cigarette smoke among adolescent girls in Canada

**DOI:** 10.1186/1471-2458-14-468

**Published:** 2014-05-17

**Authors:** Jennifer Schwartz, Raquel B Graham, Chris G Richardson, Chizimuzo T Okoli, Laura L Struik, Joan L Bottorff

**Affiliations:** 1 School of Population & Public Health, University of British Columbia, 2206 East Mall, Vancouver, BC V6T1Z3, Canada; 2 School of Nursing, University of British Columbia, 3333 University Way, Kelowna, BC V6T 1Z3, Canada; 3 Professorial Fellow, Faculty of Health Sciences, Australian Catholic University, St. Patrick’s Campus, 115 Victoria Parade, Fitzroy, Victoria 3065, Australia; 4 Tobacco Treatment and Prevention Division, University of Kentucky College of Nursing, 315 College of Nursing Building, Lexington, KY 40536, USA

**Keywords:** Second-hand smoke, Adolescents, Females, Tobacco, Risk reduction behavior

## Abstract

**Background:**

Although rates of tobacco use and exposure to second-hand smoke (SHS) are declining in Canada, SHS exposure among non-smoking adolescents remains high. This study aimed to describe frequency, locations, and avoidance behavior related to SHS exposure among adolescent girls in British Columbia, Canada.

**Methods:**

Data were analyzed from 841 adolescent girls aged 13 to 15 years old who completed an internet-delivered survey as part of a cohort study examining SHS exposure and substance use. Measures assessed demographics, smoking behavior and intentions, frequency and locations of SHS exposure, and avoidance behavior related to SHS.

**Results:**

Excluding their own smoking, 27% of girls reported exposure at least once a week and an additional 17% reported daily or almost daily exposure over the past month. Among girls who reported daily or almost daily exposure, the locations of most frequent levels of high exposure were in the home, at or near school, inside a vehicle, and outdoor public places. Avoidance behavior related to SHS exposure significantly differed by overall SHS exposure in the past month.

**Conclusions:**

Despite historically low smoking rates, many adolescent girls continue to report regular SHS exposure in multiple locations in British Columbia. Girls with the most frequent exposure were significantly less likely to report habitual avoidance behavior related to SHS compared to those less frequently exposed. This study elucidates settings of high SHS exposure among adolescent girls that could be targeted in future policy interventions. Additionally, future interventions could target adolescent girls who are frequently exposed to SHS and report infrequent avoidance behavior around their SHS exposure.

## Background

Although rates of tobacco use and exposure to second-hand smoke (SHS) are declining in Canada, SHS exposure among non-smoking adolescents remains high [1]. According to the 2007–2009 Canadian Health Measures Survey, a significantly greater percentage of non-smoking adolescents (12–19 years old) reported regular exposure to SHS compared to non-smoking children (6–11 years old) and adults (20–79 years old) [2]. In addition to poor academic performance and a greater number of school days missed due to poor health [3,4], SHS exposure within this demographic is associated with a number of negative health consequences (e.g., cancers, heart disease, ear infections, asthma, respiratory infections, and decreased pulmonary function) [5]. Among adolescents, girls are particularly vulnerable to the adverse health effects of tobacco smoke; recent research demonstrated that both active smoking and SHS exposure increase the risk for developing breast cancer, particularly when exposure occurs during puberty when breast cell proliferation is most rapid [6]. Results of a meta-analysis suggest a 60-70% increase for breast cancer risk in pre-menopausal women who report long-term SHS exposure [7]. As cigarette smoke exposure remains one of the few modifiable risk factors for breast cancer, it is essential to develop harm reduction messages and strategies for adolescent girls who are at risk of regular SHS exposure.

Emerging evidence indicates that targeted communications (e.g., by gender and/or cancer-specific) are more effective than non-targeted alternatives such as gender-neutral messaging about non-specific forms of cancer [8-10]. Adolescence, or the transformation into womanhood, is a period of marked awareness of physical changes (e.g., breast development) for girls [9]. These phases of pronounced cognizance of health transitions may increase girls’ risk perceptions, and have been recognized as teachable moments for cancer prevention interventions [11]. Therefore, targeting communications about the link between tobacco exposure and an increased risk of breast cancer to adolescent girls takes advantage of this naturally occurring teachable moment to promote reductions in SHS exposure. Additionally, there is a growing body of evidence that gender profoundly influences health behaviors and responses to teachable moments [10,11]. In fact, Richardson et al. found that gender-sensitive messages about the relationship between breast cancer and SHS increased adolescent girls’ awareness of the risks and stimulated information seeking about these risks [8].

Previous studies have investigated SHS exposure in the general population; however, few studies have identified specific locations of greatest exposure among adolescents [12]. For example, non-smoking Canadians most commonly report SHS exposure in public locations and the work place; however these findings may not extend to adolescents. Analyses from one study indicate that Canadian youth are frequently exposed to SHS in the home or vehicle; but other locations such as around schools, bus stops, and in public parks require further investigation [4].

An understanding of where adolescent girls are most exposed, as well as their efforts to avoid SHS, will provide the foundation needed to develop targeted harm reduction strategies that aim to reduce SHS exposure and ultimately breast cancer risk. Given the heightened vulnerability to carcinogens in cigarette smoke during breast development, there is a need to inform adolescent girls of their potential risk. Utilizing data from a large cohort study of adolescents in British Columbia, the present study aimed to examine frequency, specific locations, and avoidance behaviors related to SHS exposure among adolescent girls.

## Methods

### Participants

Participants were 841 adolescent girls aged 13 to 15 years who participated in an internet-based cohort study of youth in British Columbia, Canada (The BC Adolescent Substance Use Survey [BASUS]) in the spring of 2011. Students were recruited from 48 participating public secondary schools in BC, and eligibility criteria included being 13 years of age or older and the ability to read and complete the internet-based survey in English. All participants provided informed consent, as well as written parental consent in schools requiring participants to provide parental consent. All students were recruited in a school environment – after viewing a brief presentation during home room class, students were given an informational package that contained a unique login code to set up an account on the survey website – and the survey was completed online during the students’ own time or in some cases in school computer labs during scheduled class time. The school-specific response rate ranged from 2% to 100% with an average school response rate of 20%. The University of British Columbia Behavioural Research Ethics Board classified this study as minimal risk and did not require us to obtain signed parental consent. Given the age of the students and the minimal risk of the study, we were approved to use a passive consent procedure in which the take home information package included a letter to parents about the study with our contact information should they wish us to block their son/daughter from participating in the study against their wishes (i.e., we could prevent an account from being set up with their son/daughters' information package card, as well as their email address or home mailing address). This procedure was approved by the University of British Columbia Behavioral Research Ethics Board and all School Districts, as well as individual Secondary School Administrators except for one school district which has a standard policy requiring signed parental consent for all research studies involving their students. The signed parental consent procedure used in this school district was also approved by the University of British Columbia Behavioural Research Ethics Board. Data collected in the BASUS survey included participant characteristics such as age, ethnicity, family income, cigarette smoking behavior, intentions to smoke, and information on SHS exposure.

### Measurement of exposure

The online questionnaire was used to collect detailed data regarding adolescents’ exposure to SHS during the past month. The following question was used to collect data on overall SHS exposure: “Overall (excluding your own smoking) in the past month were you exposed to second-hand smoke?”, with the following response options: 1) never, 2) at least once in past month (low frequency), 3) at least once a week (medium frequency), and 4) every day or almost every day (high frequency). Additionally, the following multipart question was used to examine SHS exposure in specific locations: “In the past month (excluding your own smoking), how often were you exposed to second-hand smoke: inside a car or other vehicle?; inside someone else’s home?; on an outdoor patio of a restaurant or bar?; at a bus stop or shelter?; at an entrance to a building?; at your workplace?; at/near your school?; at any other public place such as a shopping mall, arena, concert, or sporting event?; and/or outdoors such as on a sidewalk or in a park?”. The presence of home cigarette smoking restrictions was assessed with the following yes/no question: “Are there any restrictions against smoking cigarettes in your home?”

### Stage of change related to avoidance of SHS

A brief measure, which was developed based on the Prochaska’s Stage of Change model on an adolescent sample [13,14], was used to examine avoidance behavior related to SHS exposure. Specifically, the measure assessed adolescents’ stage of change (i.e., maintenance, action, preparation, contemplation and pre-contemplation) related to reducing exposure to SHS with the following question:

When you are exposed to second-hand cigarette smoke do you consistently do things to reduce your exposure to the smoke? (Please check only one)

1) *Yes, I have been for more than 6 months (MAINTENANCE).*

2) *Yes, I have been, but for less than 6 months (ACTION).*

3) *No, but I intend to in the next 30 days (PREPARATION).*

4) *No, but I intend to in the next 6 months (CONTEMPLATION).*

5) *No, and I do NOT intend to in the next 6 months (PRE-CONTEMPLATION).*

Based on responses to this question, adolescents were categorized as being in a particular stage with regard to avoidance behavior related to SHS exposure.

### Statistical analysis

Descriptive statistics of the sample are provided, and a bivariate analysis (Pearson Chi-square test) was performed to examine the relationship between overall SHS exposure and risk reduction behavior around SHS in the past month. An alpha level of p < 0.05 (2-tailed) was used to indicate statistical significance. All statistical analyses were conducted using IBM SPSS Statistics 19.0. A series of Venn diagrams were created to evaluate the extent to which environmental and social variables are related to high levels of SHS exposure in three of the most commonly reported exposure settings. Among adolescents who reported high SHS exposure in the home (n = 130), we were specifically interested in understanding how many also had parents who smoke and reported home smoking restrictions. Among those who reported high SHS exposure inside a vehicle (n = 96), we were interested in examining how many also had friends or parents who smoke. Finally, among adolescents who reported high SHS exposure at school and other outdoor locations (n = 88), we evaluated how many adolescents also had friends who smoke. A 95% confidence interval was used to provide a range of plausible values for these parameters, and to investigate the likelihood of specific exposure groups overlapping with the larger exposure group of interest.

## Results

Table 1 displays the adolescent girls’ demographics, smoking behavior and intentions, and reported levels of SHS exposure. Fifty-seven percent of girls were 14 years old, 69% were in grade nine, 50% were Caucasian, and 78% reported an average family income. Most of the girls had never tried smoking (88%) and reported that they definitely did not intend on smoking in the future (75%); of those girls who had tried smoking, the majority were not current smokers (60%) and were 13 years of age or older when they tried their first cigarette. Moreover, a majority of girls reported that they did not have parents (68%) or friends (80%) who smoke, and had home smoking restrictions (87%).

**Table 1:** Characteristics of adolescent girls (N = 841) in a study on second-hand smoke (SHS) exposure

***Demographics***	
Age	
*13 years*	144 (17.1%)
*14 years*	479 (57.0%)
*15 years*	218 (25.9%)
Grade	
*8*	251 (30.0%)
*9*	578 (69.0%)
*10*	8 (1.0%)
Ethnicity	
*Caucasian*	419 (50.1%)
*Aboriginal*	111 (13.3%)
*Asian*	286 (34.2%)
*Other*	21 (2.5%)
Family Income (self-reported)	
*Below average*	46 (5.9%)
*Average*	611 (78.0%)
*Above average*	126 (16.1%)
***Smoking behavior and intentions***	
Ever tried smoking	
*Yes*	97 (11.5%)
Current smoker (smoked ≥once in past 30days); among those who indicated they had ever tried smoking (N=97)	
*Yes*	38 (40%)
Age at first cigarette; among those who indicated they had ever tried smoking (N=97)	
*≤12 years*	39 (41.5%)
*13 years*	29 (30.9%)
*≥14 years*	26 (27.7%)
Smoking intentions	
*Definitely/probably yes*	28 (3.9%)
*Probably not*	153 (21.3%)
*Definitely not*	539 (74.9%)
***SHS exposure***	
Parents smoke	
*Yes*	236 (31.7%)
Friends smoke	
*Yes*	128 (20.3%)
Home smoking restrictions	
*Yes*	692 (87.0%)

As shown in Table 2, overall SHS exposure varied, with 17% (139/841) of the sample reporting exposure every day or almost every day, and 27% (223/841) reporting exposure at least once a week. Reports of any SHS exposure in various smoking locations among girls with at least weekly overall exposure were as follows: 32% (115/358) in the home, 44% (159/360) in a vehicle or car, 48% (172/359) in someone else’s home, 86% (311/360) at/near school, 75% (267/358) on an outdoor patio of a bar or restaurant, 77% (277/359) at a bus stop or shelter, 93% (331/357) outdoors on sidewalk or park, 89% (319/359) at an entrance to a building, and 91% (323/357) at any other public place such as a shopping mall, arena, concert, or sporting event. Patterns of exposure varied by location, particularly among high SHS exposure groups. For example, among adolescent girls who reported exposure every day or almost every day, 39% (95%CI: 30.8-47.5) reported in home exposure every day or almost every day. Similarly, among those who reported exposure every day or almost every day, 34% (95%CI: 26.2-42.4) reported being exposed ‘a lot’ at or near school.

**Table 2:** Frequency of second-hand smoke exposure at specific locations among adolescent girls with at least weekly overall exposure in the past month (N = 362)

**Overall (excluding your own smoking) in the past month were you exposed to second hand smoke?**		**At least once a week (N = 223) % (95% CI)**	**Every day or almost every day (N = 139) % (95% CI)**
**In the past month (excluding your own smoking), how often were you exposed to SHS:**			
In your own home	Never (n = 243)	80% (73.8-84.8)	48% (38.3-55.4)
	At least once in past month (n = 24)	7% (4.0-11.1)	7% (3.2-12.3)
	At least once a week (n = 29)	10% (6.1-14.2)	6% (2.7-11.4)
	Every day or almost every day (n = 62)	4% (1.7-7.2)	39% (30.8-47.5)
Inside a car or other vehicle	Never (n = 201)	67% (59.7-72.5)	38% (30.1-46.8)
	Once (n = 39)	12% (7.9-16.8)	9% (5.3-15.8)
	A few times (n = 54)	15% (10.9-20.8)	14% (9.2-21.6)
	More than a few times (n = 28)	4% (1.7-7.2)	14% (9.2-21.6)
	A lot (n = 38)	2% (0.8-5.4)	24% (17.1-31.8)
Inside someone else’s home	Never (n = 187)	59% (51.5-64.8)	41% (32.8-49.7)
	Once (n = 57)	17% (12.1-22.3)	15% (9.2-21.6)
	A few times (n = 70)	19% (13.7-24.2)	21% (14.6-28.7)
	More than a few times (n = 22)	4% (1.7-7.2)	10% (5.8-16.6)
	A lot (n = 23)	2% (0.8-5.4)	13% (8.1-20.0)
At/near your school	Never (n = 49)	15% (10.9-20.8)	11% (6.4-17.5)
	Once (n = 40)	14% (9.4-18.8)	7% (3.7-13.2)
	A few times (n = 131)	40% (33.1-46.2)	31% (23.5-39.4)
	More than a few times (n = 59)	16% (11.7-21.8)	17% (11.0-24.0)
	A lot (n = 81)	15% (10.9-20.8)	34% (26.2-42.4)
On an outdoor patio of a restaurant or bar	Never (n = 91)	26% (20.1-31.9)	25% (17.8-32.6)
	Once (n = 46)	17% (12.1-22.3)	7% (3.2-12.3)
	A few times (n = 136)	40% (33.5-46.7)	34% (26.2-42.4)
	More than a few times (n = 48)	12% (7.9-16.8)	16% (10.4-23.2)
	A lot (n = 37)	5% (2.9-9.4)	18% (12.2-25.6)
At a bus stop or shelter	Never (n = 82)	20% (15.2-26.2)	27% (19.7-34.9)
	Once (n = 38)	11% (7.5-16.3)	10% (5.3-15.8)
	A few times (n = 133)	44% (36.9-50.3)	27% (19.0-34.1)
	More than a few times (n = 57)	18% (13.3-23.8)	13% (7.5-19.1)
	A lot (n = 49)	7% (4.3-11.6)	24% (17.1-31.8)
Outdoors such as on a sidewalk or in a park	Never (n = 26)	5% (2.6-8.9)	11% (6.4-17.5)
	Once (n = 35)	15% (10.2-19.8)	2% (0.6-6.7)
	A few times (n = 136)	44% (36.5-49.8)	29% (21.6-37.2)
	More than a few times (n = 87)	22% (16.8-28.1)	28% (20.3-35.7)
	A lot (n = 73)	15% (10.2-19.8)	30% (22.2-37.9)
At an entrance to a building	Never (n = 40)	11% (7.2-15.8)	12% (6.9-18.3)
	Once (n = 38)	11% (7.2-15.8)	10% (5.8-16.6)
	A few times (n = 148)	47% (40.0-53.4)	32% (24.2-40.2)
	More than a few times (n = 80)	23% (18.1-29.5)	21% (14.0-28.0)
	A lot (n = 53)	9% (5.3-13.2)	25% (17.8-32.6)
At any other public place such as a shopping mall, arena, concert, or sporting event	Never (n = 34)	9% (5.3-13.2)	11% (6.4-17.5)
	Once (n = 23)	9% (5.7-13.7)	2% (0.6-6.7)
	A few times (n = 156)	49% (41.7-55.2)	35% (26.8-43.1)
	More than a few times (n = 80)	23% (17.6-29.1)	21% (14.6-28.7)
	A lot (n = 64)	10% (6.1-14.2)	31% (23.5-39.4)

**Figure 1:**
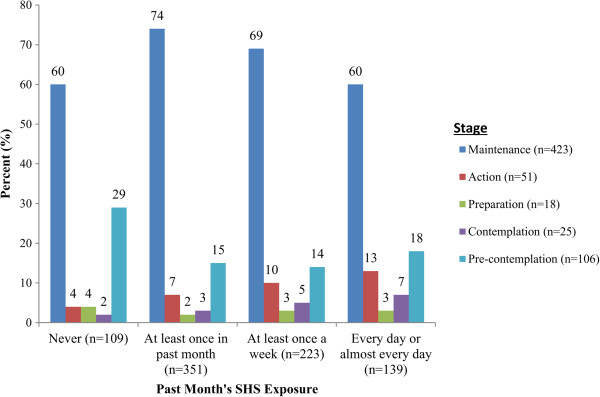
**Stage of change related to avoidance behavior around SHS by frequency of SHS exposure in the past month in adolescent girls (n = 841).** *Based on chi-square test, p < 0.001. *Based on chi-square test, p < 0.001.

Figure 1 illustrates the adolescent girls’ stage of change with regard to avoidance behavior around SHS by overall SHS exposure in the past month. For example, 18% and 14% of adolescent girls who were exposed to SHS every day or almost every day, and at least once a week, respectively, were in the pre-contemplation stage with regard to avoidance behavior around SHS. Additionally, 15% of girls who were exposed at least once in the past month were in the pre-contemplation stage. Based on chi-square analysis, stage of change with regard to SHS risk reduction behavior significantly differed by overall SHS exposure in the past month among adolescent girls (p < 0.001).

**Figure 2:**
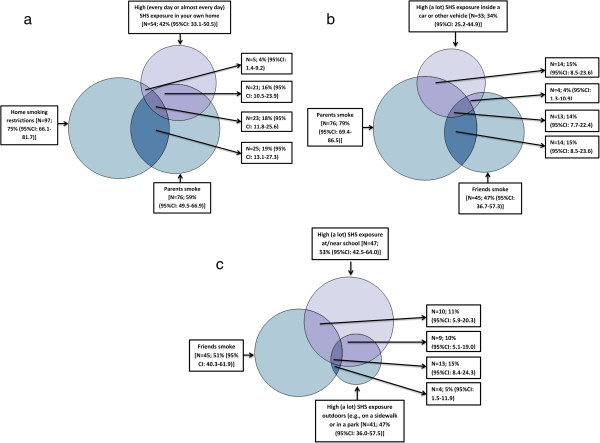
**Venn diagrams displaying SHS exposure locations, sources, and related variables among adolescent girls with high overall SHS exposure in the past month (N = 139). Panel a.** In-home Exposure (N=130). **Panel b.** Car Exposure (N=96). **Panel c.** Outdoors – At/near school and on sidewalks or in parks (N=88).

A series of Venn diagrams were generated to explore patterns of SHS exposure in multiple locations, and the extent to which other factors (e.g., having parents who smoke) might overlap with reporting SHS exposure in particular locations. We were specifically interested in gaining insight into the extent that high SHS exposure in the home overlapped with reporting home smoking restrictions and/or having parents who smoke. We were also interested in examining the extent to which high SHS exposure in a vehicle coincided with having parents who smoke and/or friends who smoke. Finally, we examined the overlap between those who reported having friends who smoke and those who reported high SHS exposure at/near school and outdoors (on a sidewalk or in a park). As displayed in Panel 1 of the Venn diagrams (Figure 2), among girls with both high (every day or almost every day) overall SHS exposure in the past month and complete data on in-home exposure (n = 130), 42% (95% CI: 33.1-50.5) also had high SHS exposure in the home; 59% (95% CI: 49.5-66.9) had parents who smoke; and 74% (95% CI: 66.1-81.7) had home smoking restrictions. Furthermore, among these highly exposed girls with complete data on in-home exposure, 4% (95% CI: 1.4-9.2) had high SHS exposure in the home and reported home smoking restrictions; 16% (95% CI: 10.5-23.9) had high SHS exposure in the home and had parents who smoke; 19% (95% CI: 13.1-27.3) had parents who smoke and home smoking restrictions; and an additional 18% (95% CI: 13.1-27.3) had high SHS exposure in the home, home smoking restrictions, and parents who smoke.

As displayed in Panel 2 of the Venn diagrams (Figure 2), among girls with both high (every day or almost every day) overall SHS exposure in the past month and complete data on SHS exposure in cars (n = 96), 34% (95% CI: 25.2-44.9) also had high SHS exposure inside a car or other vehicle; 79% (95% CI: 69.4-86.5) had parents who smoke; and 47% (95% CI: 36.7-57.3) had friends who smoke. Furthermore, among these highly exposed girls, 15% (95% CI: 8.5-23.6) had high SHS exposure inside a car or other vehicle and had parents who smoke; 4% (95% CI: 1.3-10.9) had high SHS exposure inside a car or other vehicle and had friends who smoke; and an additional 14% (95% CI: 7.7-22.4) had high SHS exposure inside a car or other vehicle, friends who smoke, and parents who smoke.

As displayed in Panel 3 of the Venn diagrams (Figure 2), among girls with both high (every day or almost every day) overall SHS exposure in the past month and complete data on SHS exposure outdoors (at/near school and on sidewalks or in parks) (n = 88), 53% (95% CI: 42.5-64.0) also had high SHS exposure at or near school; 51% (95% CI: 40.3-61.9) had friends who smoke; and 47% (95% CI: 36.0-57.5) had high SHS exposure outdoors. Furthermore, among these highly exposed girls, 11% (95% CI: 5.9-20.3) had high SHS exposure at or near school and had friends who smoke; 10% (95% CI: 5.1-19.0) had high SHS exposure at or near school and had high SHS exposure outdoors; and an additional 15% (95% CI: 8.4-24.3) had high SHS exposure at or near school, friends who smoke, and high SHS exposure outdoors. Relationships displayed in the Venn diagrams are also shown in a table for ease of interpretation (see Additional file 1: Table S1).

## Discussion

To our knowledge, this is the first study to describe the reported frequency, locations, and avoidance behaviors related to SHS exposure among adolescent girls in Canada. We found that although the majority of adolescent girls in our sample had never tried smoking, a concerning proportion reported regular exposure to SHS. Of the 841 girls, 139 (17%) reported exposure to SHS every day or almost every day in the past month, and an additional 223 (27%) reported exposure at least once a week. Within this group, regular exposure frequently occurred in the home, at/near school, inside a vehicle, at bus stops, and other public locations. Of note, the in-home SHS exposure rate (19%) reported by this cohort of adolescent girls is consistent with recent research by Healey et al. on youth in New Zealand aged 14 to 15 years, who reported that in-home SHS exposure rates varied from 12% among youth with no parents who smoked, to 85% among youth with both parents who smoked [15].

Avoidance behavior around SHS was examined using a brief measure adapted from the stage of change model [13]. Compared to girls who reported less SHS exposure, girls who were exposed every day or almost every day were more likely to be in the pre-contemplation, contemplation, or preparation stages. However, 60% of girls who were exposed every day or almost every day were in the maintenance stage, suggesting that the majority of girls with regular SHS exposure consistently take action to reduce their exposure. It is possible that greater SHS exposure prompts avoidance behaviors, although motivational factors underlying efforts to avoid SHS were not assessed in this study. Future studies may explore strategies girls implement to reduce SHS exposure and evaluate the effectiveness of such strategies. Furthermore, social norms may influence girls’ perceived ability to avoid SHS. Therefore, interventions that address social pressures that undermine avoidance of SHS could be created.

The Venn diagrams (Figure 2), which display SHS exposure locations, sources, and related variables among adolescent girls with high overall SHS exposure in the past month, provide interesting findings. For example, despite reporting home smoking restrictions, 22% of adolescent girls reported being exposed in their home every day or almost every day. Meanwhile, as displayed in the second Venn diagram (Panel 2), only 28% of girls who reported having parents who smoke also reported high SHS exposure inside a car or other vehicle. As shown in the third Venn diagram (Panel 3), the majority of girls who reported being regularly exposed at/near school did not actually have friends who smoke, suggesting they were exposed in areas where smoking occurs among a larger group of peers. These findings have important implications for informing the development of risk-reduction messages and/or interventions, and demonstrate the importance of targeting such messages/interventions to specific populations and/or locations. For example, messages could target locations where adolescent girls are frequently exposed, such as bus stops and at/near schools. Moreover, interventions could target individuals in the pre-contemplation and/or contemplation stages with the goal of shifting them into the action or maintenance stages related to avoidance of SHS.

The current findings also highlight the need to educate parents about the importance of enforcing complete home smoking bans and protecting girls from SHS exposure. With increasing prohibitions on smoking in public places, the home is becoming a primary source of SHS exposure [16]. This is reflected in our findings – 39% of the girls with high overall SHS exposure reported being exposed every day or almost every day in their home. This is especially concerning in light of evidence that particulate matter from tobacco smoke in homes where smoking occurred were at unhealthy levels even in remote areas of the home where smoking does not typically occur [17]. Given that adolescents may be limited in influencing home smoking restrictions, it is important that parents are educated about the dangers of SHS exposure in the home. Information about SHS and breast cancer could provide the basis for a renewed message to parents about the importance of smoke-free homes, especially for adolescent girls.

Our findings offer important implications for policy makers. Despite prohibitions on smoking in most public places since 2008 in British Columbia [16], the girls in our study reported regular SHS exposure in several of the locations where these bans are in place. For example, of those most exposed to SHS, 34% reported regular exposure at or near school, 25% at an entrance to a building, 24% inside a car or other vehicle, and 18% on an outdoor patio of a restaurant or bar. These findings highlight the importance of ensuring the development, implementation, and enforcement of well-defined policies in relation to smoking prohibitions in these places, in order to create environments that protect adolescents rather than placing the burden on them to avoid SHS.

This study is not without limitations. British Columbia has a strong history of tobacco control, which has resulted in decreased smoking rates and policies restricting smoking in public spaces. Levels of SHS exposure and efforts to avoid SHS are likely to be higher in other regions where smoking rates remain high. It is also possible that the order in which the questions were presented may have influenced individual responses. Participants were first asked about their efforts to reduce exposure, followed by questions about specific locations of exposure, and finally about their overall smoke exposure during the past month. Priming individuals to think about exposure in specific locations may have influenced responses to the overall exposure question. Current smokers, although more likely to associate with other smokers and less likely to engage in SHS avoidance behavior, were included in analyses to reduce the likelihood of biasing the sample towards less SHS exposure than expected in a typical Canadian school. Although the average participation rate within schools was relatively low, the representativeness of our sample is supported by findings that indicate a smoking rate of 4.5% among adolescents in grades 8–10, which is similar to results based on the nationally representative Youth Smoking Survey (2010–11), which indicated a 2% and 10% prevalence of current smokers among youth in grades 6–9 and 10–12, respectively [18]. Furthermore, the finding that 7.4% of girls in this study reported every day or almost every day exposure in the home is similar to findings from the 2011 Canadian Tobacco Use Monitoring Survey which found that 7.1% of Canadian children aged 12–17 years were regularly exposed to tobacco smoke in their homes [19]. It should also be noted that 78% of our sample reported an average family income, which may have resulted from social desirability bias. Nonetheless, we acknowledge that this relatively high proportion potentially limits generalizability of our findings to individuals in other socioeconomic groups. Lastly, the descriptive data displayed in the Venn diagrams should be interpreted with consideration for the small sample sizes.

### Future research

The findings from this study suggest the need for further research among adolescent girls in other regions to assess the influence of smoking rates and policies restricting smoking in public spaces on girls’ SHS exposure. From a methodological perspective, although researchers have validated adolescents’ self-reported smoking status [20], research is needed to validate the self-reported SHS exposure measures used in this study. Since youth may be in situations where they have limited influence on the smoking behaviors of adults around them, it is recommended to evaluate approaches to inform parents and others who smoke regarding the specific health risks of SHS exposure for adolescent girls. In addition, an exploration of adolescent girls’ efforts to avoid SHS exposure and the effectiveness of their strategies could inform interventions to promote and support girls and young women in their efforts to avoid SHS.

## Conclusions

SHS exposure among adolescent girls in British Columbia remains high despite many smoking restrictions in public spaces. This level of exposure is especially concerning given the recent evidence demonstrating an associated increased risk of breast cancer [7]. Compared to girls who reported less frequent exposure, those who reported the most exposure were significantly less likely to report avoidance behavior around SHS. Developing approaches to inform both smokers and those exposed to SHS about the specific SHS exposure risk to young women is important. Interventions targeting adolescent girls with frequent SHS exposure who are in the pre-contemplation and/or contemplation stages in relation to SHS risk reduction behavior are needed to reduce exposure and ultimately breast cancer risk.

## Competing interests

The authors declare that they have no competing interests.

## Authors’ contributions

JS was involved in conceptualizing and designing the study, conducting the statistical analyses, and drafting the manuscript. CG (Co-PI of the START study) was involved in conceptualizing and designing the study, participated in the statistical analyses, and revised the manuscript for intellectual content. RG was involved in drafting the manuscript, creating figures, and revising the manuscript. CO was involved in conceptualization of the study. LS was involved in drafting and revising the manuscript. JB (PI of the START study) was involved in conceptualization, design, and revising the manuscript. All authors read and approved the final manuscript.

## Pre-publication history

The pre-publication history for this paper can be accessed here:

http://www.biomedcentral.com/1471-2458/14/468/prepub

## Supplementary Material

Additional file 1: Table S1 Sources and related variables of SHS exposure by specific exposure locations among adolescent girls with high overall SHS exposure (n = 139). Click here for file
