# Supplementary material for: An examination of exposure and avoidance behavior related to second-hand cigarette smoke among adolescent girls in Canada
Source: BMC Public Health. 2014 May 17;14:468. doi: 10.1186/1471-2458-14-468 (PMC4037430; doi:10.1186/1471-2458-14-468)
Supplement: Additional file 1: Table S1 — Sources and related variables of SHS exposure by specific exposure locations among adolescent girls with high overall SHS exposure (n = 139). [file 1471-2458-14-468-S1.doc]

Additional file 1: Table S1. Sources and related variables of SHS exposure by specific exposure locations among adolescent girls with high overall SHS exposure (n=139).

|  | **High (every day or almost every day) SHS exposure in your own home (N=54)** | **High (a lot) SHS exposure inside a car or other vehicle (N=33)** | **High (a lot) SHS exposure at/near school (N=47)** | **High (a lot) SHS exposure outdoors (e.g., on a sidewalk or in a park)(N=41)** |
| --- | --- | --- | --- | --- |
| Home smoking restrictions | *Yes* (N=28, 52%) |  |  |  |
| Parents smoke | *Yes* (N=44, 81%) | *Yes* (N=27, 82%) |  |  |
| Friends smoke |  | *Yes* (N=17, 52%) | *Yes* (N=23, 49%) | *Yes* (N=17, 41%) |
| High SHS exposure outdoors |  |  | *Yes* (N=22, 47%) |  |
